# The effectiveness of land based exercise compared to decompressive surgery in the management of lumbar spinal-canal stenosis: a systematic review

**DOI:** 10.1186/1471-2474-13-30

**Published:** 2012-02-28

**Authors:** Mark S Jarrett, Joseph F Orlando, Karen Grimmer-Somers

**Affiliations:** 1 International Centre for Allied Health Evidence, University of South Australia, North Terrace, Adelaide, SA 5000, Australia; 2 Eastwood Physiotherapy, Fullarton Road, Eastwood, SA 5063, Australia; 3 Physiotherapy Department, Royal Adelaide Hospital, North Terrace, Adelaide, SA 5000, Australia

## Abstract

**Background:**

Lumbar spinal stenosis (LSS) is prevalent in those over the age of 65 years and the leading cause of spinal surgery in this population. Recent systematic reviews have examined the effectiveness of conservative management for LSS, but not relative to surgical interventions. The aim of this review was to systematically examine the effectiveness of land based exercise compared with decompressive surgery in the management of patients with LSS.

**Methods:**

A systematic review of randomised controlled trials and clinical trials was undertaken. The databases MEDLINE, Embase, CINAHL, PEDro and Cochrane Library Register of Controlled Trials were searched from January 2000 to June 2011. Only studies that included subjects with lumbar spinal canal stenosis were considered in this review. Studies also had to use a patient reported functional outcome measure for a land based exercise intervention or lumbar decompressive surgery.

**Results:**

Only one study compared the effectiveness of exercise and decompressive surgery for LSS. Surgery demonstrated statistically significant improvements in patient reported functional outcome scores at 6, 12 and 24-months post-intervention (*p *< 0.01). To facilitate further analysis, the results from 12 exercise and 10 surgical intervention arms were compared using percentage change in patient reported functional outcome measure scores. Exercise interventions showed initial improvements, ranging from 16 to 29% above baseline. All decompressive surgical interventions demonstrated greater and sustained improvements over 2-years (range 38-67% improvement) with moderate to large effect sizes. The most commonly reported complications associated with surgery were dural tears, while details of adverse effects were lacking in exercise interventions.

**Conclusions:**

This systematic review of the recent literature demonstrates that decompressive surgery is more effective than land based exercise in the management of LSS. However, given the condition's slowly progressive nature and the potential for known surgical complications, it is recommended that a trial of conservative management with land based exercise be considered prior to consideration of surgical intervention.

## Background

Degenerative lumbar spinal stenosis (LSS) is a prevalent condition with 47% of people between 60 and 69 years demonstrating radiographic findings [1]. Whilst the incidence of people who develop symptoms is unknown, LSS is the leading cause of spinal surgery in adults greater than 65 years of age [2]. LSS can impact a person's quality of life, lead to activity and participatory restrictions and affect psychosocial wellbeing. Managing these health impacts and therefore improving health status becomes a primary goal of self-management [3].

There has been a growing movement towards self-management of chronic conditions in the past decade, including from policy makers due to the increasing cost and utilisation of healthcare resources from an ageing population and rising incidence of chronic conditions [4]. The understanding of pain and stress sciences has also developed this past decade [5,6] and helped to explain the influences of psychosocial variables in a pain or stress experience. Such variables may potentially explain the poor correlation between LSS radiographic and clinical findings [7]. Given that LSS is a slow to progress, degenerative condition and commonly sought for surgical opinion [2], it seems appropriate that self-management options be offered, which may include physiotherapy.

Exercise is a core component of physiotherapy that promotes self-management. It aims to improve the flexibility or mobility of the spine and combats both physical and psychological effects of deconditioning associated with pain and functional restrictions. Exercise based interventions can be delivered in a low cost manner and continued as a home program [8-10].

There have been a number of recent systematic reviews that have examined the effectiveness of exercise for LSS with mixed results. Reiman and colleagues [11] and Iversen and colleagues [12] reported preliminary evidence that manual therapy when combined with exercise therapy is of potential benefit in LSS, while Watters and colleagues [13] concluded that there was insufficient evidence for exercise. The latter provided additional evidence, albeit low level and poor quality, to support lumbar surgery over conservative management, including physiotherapy and medical intervention. Despite this, formal comparison of exercise and decompressive surgery has not been undertaken. Although there is a limited evidence base, in clinical practice it appears that a trial of self-management is preferred prior to surgical interventions to avoid the risks associated with surgery [10,14,15].

In light of the potential benefits of exercise for LSS, we systematically reviewed the current evidence regarding the effectiveness of land based exercise interventions compared to surgical decompression in the management of LSS. The secondary aim of this review was to report on the adverse effects associated with the use of these two interventions.

## Methods

### Search strategy

This review was reported in line with the PRISMA Statement [16]. The databases MEDLINE, Embase, CINAHL, PEDro and Cochrane Library Register of Controlled Trials were searched from January 2000 to June 2011. The limit to recent years intended to capture interventions in line with the development of self-management models. Table 1 outlines the search question and the keywords used in the search strategy. The reference list of all included studies were also searched for any relevant studies not located by the electronic search.

**Table 1:** Search strategy (PECOT) and search terms

	Definition	Search terms
Population	Adults with degenerative LSS	lumbar stenosis or spinal stenosis or canal stenosis or vertebral stenosis

Exposure	Land based exercise programs	exercise or non-operative management or conservative management or therapeutic exercise or physiotherap* or physical therap* or flexibility or range of movement or range of motion or stretching or aerobic exercise

Comparator	Decompressive surgery	surgery or surgical or laminectomy or decompression or operative management or operation

Outcome	Patient reported functional outcome measure for low back pain	As described in the study

Timeframe	Follow-up within 2-years of intervention	As described in the study

### Study design

All prospective experimental studies, which described measurements taken pre and post-interventions for exercise and/or surgical decompression were considered for inclusion. Secondary evidence such as systematic reviews and retrospective studies were excluded. The reference lists of systematic reviews were searched for additional studies for inclusion.

### Population

Studies were only considered if they included subjects with degenerative LSS as diagnosed by MRI/CT and clinical presentation. Symptoms typically include buttock or lower extremity pain with or without back pain, aggravated by lumbar extension and ambulation and relieved by lumbar flexion and sitting [13]. Studies that included subjects with a concurrent diagnosis of spondylolisthesis or foraminal stenosis were excluded, as were studies that investigated subjects with non-specific low back pain. Studies were also excluded if subjects were diagnosed with other spinal conditions, including congenital stenosis, fracture, infection, tumour, inflammatory disease or osteoporosis.

### Exposure

Studies were included if one of the intervention arms was land based exercise or decompressive surgery. Studies were only included if they specified the exercise type such as flexibility, range of movement, strengthening and/or general conditioning. Aquatic exercises were excluded. Studies that combined other forms of conservative intervention, such as manual therapy, electrotherapy or medication, were included provided an exercise intervention was also undertaken.

For surgical interventions, only studies where the surgical aim was decompression of neurovascular structures in the lumbar spinal canal, such as laminectomy, were included. Minimally invasive approaches to decompressive surgery were also included. Studies that incorporated lumbar fusion for management of pre-operative instability (spondylolisthesis) were excluded, as were studies that did not describe surgical intervention.

### Outcome measures

Studies were included if they utilised a patient reported functional outcome measure for low back pain, such as Oswestry Disability Index (ODI) or Roland-Morris Disability Questionnaire (RMQ).

### Time

Studies were included if they reported an outcome within 2-years of completing the intervention. Studies that only investigated outcomes with follow-up periods greater than 2-years were excluded to limit the impact of confounding variables associated with longer-term follow-up. Studies were also excluded if outcome data for all subjects was not collected at predetermined timeframes.

### Search of literature

After establishing a review protocol, two authors (MJ, JO) divided the database searches and screening of titles and abstracts. Studies were excluded if they were published in languages other than English. Whilst this may present a source of publication bias, the evidence to support this is in the literature is equivocal [17,18]. All potentially included abstracts and full texts were reviewed independently by the authors if studies appeared to meet the inclusion criteria. Disagreement regarding inclusion was resolved through discussion.

### Methodological quality

The methodological quality of studies was assessed using the McMaster Critical Review Form for Quantitative Studies [19]. The tool allows for the critical appraisal of studies of different quantitative study designs. It comprises 15 items that assess study purpose, design, sample, outcomes, interventions, results and clinical implications. Fourteen of the items allow o for 'yes', 'no', 'not addressed' or 'not applicable' responses, while the remaining item is a descriptive assessment pertaining to type of study design. Of the 14 quantitative criteria, the authors awarded one point for each 'yes' response and zero points for 'no' or 'not addressed' responses and calculated a raw score. 'Not applicable' responses were omitted. The raw score was expressed as a percentage of fulfilled criteria.

The hierarchy of evidence for each study was assessed according to the National Health and Medical Research Council (NHMRC) Designation of Levels of Evidence [20]. The authors (MJ, JO) independently appraised the quality of the included articles. Discrepancies in scores were resolved through discussion.

### Data extraction and analysis

Relevant information was extracted from the studies by two authors (MJ, JO) working collaboratively. Data included study design, inclusion/exclusion criteria, sample characteristics (sample size, mean age, gender), intervention details, outcome measures, follow-up periods and results. Ninety-five per cent confidence intervals were calculated for mean age of intervention arms. This enabled examination of homogeneity of age between intervention arms.

Percentage change in outcome scores was calculated for individual intervention arms to facilitate comparison between surgery and exercise. This was calculated by dividing the change in outcome scores by the baseline outcome score, then multiplied by 100 [21]. Effect sizes of interventions were calculated where possible (mean difference of intervention and control divided by the standard deviation of the control at baseline) for intervention versus control studies or exercise versus decompressive surgery studies. Strength of effect sizes is reported as small (< 0.4), moderate (0.4-0.8) and strong (≥ 0.8) [22].

## Results

**Figure 1:**
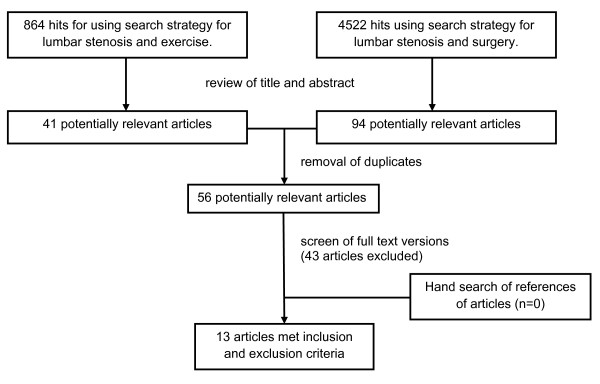
**CONSORT flowchart**.

The study selection process is summarised in Figure 1. From a review of titles and abstracts, 56 potentially relevant studies were identified from the electronic search. No further studies were identified from reference lists of systematic reviews.

### Excluded studies

Of these 56 studies, 13 fulfilled the inclusion and exclusion criteria. Studies excluded from the review are presented in a table in Additional file 1. The main reasons for exclusion were:

• outcomes were not patient reported functional outcome measures (n = 12);

• non-experimental research design (n = 10);

• populations with lumbar pathological diagnoses other than degenerative LSS (n = 9);

• recording outcomes at variable timeframes or at greater than 2-years post intervention (n = 6);

• insufficient details of intervention to allow comparison with other studies (n = 4); and

• languages other than English (n = 2).

### Methodological quality

The included studies were generally of moderate methodological quality. The McMaster Critical Appraisal Tool raw scores varied from 8 to 12 out of a possible 15, and percentages from 64.3% to 85.7% (mean 73.7%, SD 7.1). There were no discrepancies between the authors. A summary of the critical appraisal scores is provided in Table 2.

**Table 2:** Methodological quality of studies based on the McMaster Critical Appraisal Tool [19]

	Item															Score	
**Authors**	**1**	**2**	**3**	**4**	**5**	**6**	**7**	**8**	**9**	**10**	**11**	**12**	**13**	**14**	**15**	**Raw**	**%**
**Level II**																	
Goren et al. 2010	✓	✓	RCT	✓	x	?	?	✓	✓	✓	✓	✓	✓	✓	✓	12	85.7
Koc et al.2009	✓	✓	CT	✓	x	?	?	✓	✓	?	✓	✓	✓	✓	✓	10	71.4
Malmivaara et al. 2007	✓	✓	CT	✓	✓	?	?	✓	x	x	✓	✓	✓	✓	✓	10	71.4
Pua et al. 2007	✓	✓	CT	✓	✓	?	?	✓	✓	?	✓	✓	✓	✓	✓	11	78.6
Thome et al. 2005	✓	✓	CT	✓	x	?	✓	✓	✓	x	✓	✓	✓	✓	✓	11	78.6
Weinstein et al. 2008	✓	✓	RCT	✓	✓	?	?	✓	x	x	✓	✓	✓	✓	✓	10	71.4
Whitman et al. 2006	✓	✓	CT	✓	✓	?	?	✓	✓	✓	✓	✓	✓	✓	✓	12	85.7
**Level III-1**																	
Sahin et al.2009	✓	✓	C	✓	x	?	?	✓	✓	x	✓	✓	✓	x	✓	9	64.3
**Level III-2**																	
Athiviraham & Yen 2007	✓	✓	C	✓	✓	?	?	✓	✓	?	✓	✓	✓	✓	✓	11	78.6
Sobottke et al. 2010	✓	✓	C	✓	x	?	?	✓	x	?	✓	✓	✓	✓	✓	9	64.3
**Level III-3**																	
Cavusoglu et al. 2007	✓	✓	AB	✓	x	?	?	✓	n/a	x	✓	✓	✓	✓	✓	9	69.2
Chopko & Caraway 2010	✓	✓	AB	✓	x	?	?	✓	n/a	?	✓	✓	✓	✓	✓	9	69.2
Yasar et al. 2009	✓	✓	AB	✓	x	?	?	✓	n/a	x	✓	✓	✓	✓	✓	9	69.2

✓ = yes; x = no; ? = not addressed; n/a = not applicable; AB = before and after; RCT = randomised controlled trial; CT = randomised clinical trial; C = prospective cohort

McMaster Items: 1. study purpose clearly stated; 2. background literature reviewed; 3. research design; 4. sample described in detail; 5. sample size justified; 6. outcome measure reliability reported; 7. outcome measure validity reported; 8. intervention described; 9. contamination avoided; 10. co-intervention avoided; 11. results reported in terms of statistical significance; 12. analysis methods appropriate; 13. clinical significance reported; 14. drop-outs reported; 15. conclusions appropriate.

Level of evidence based on NHMRC Designation of Levels of Evidence [20].

Of the 13 included studies (see Table 2), there were two randomised controlled trials and five randomised clinical trials (Level II), one pseudo-randomised trial (Level III-1), two prospective cohort trials (Level III-2) and three before-and-after trials (Level III-3) [20]. Common aspects affecting the studies' quality included not reporting reliability and validity of outcome measures, sample sizes not well justified and of those that did calculate sample size, the studies were commonly under powered [10,23-25]. Most studies reported drop-out rates; however intention to treat analysis was rarely reported.

### Study characteristics

**Figure 2:**
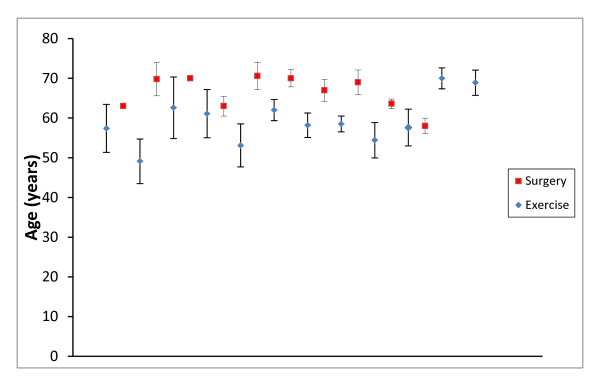
Mean age (± 95% CI*) for each intervention arm. *CIs not calculable for two intervention arms [23,30].

Six studies were identified that included a land based exercise intervention [10,24-28]. As detailed in Table 3, 12 relevant exercise arms were identified. The mean age of subjects who participated in an exercise intervention was 59.4 (SD 6.1) years. Eight studies were identified that included decompressive surgery [10,23,29-34]. Table 3 also summarises the ten decompressive surgical intervention arms identified. The mean age of subjects in surgical interventions was 66.4 (SD 4.27) years. Comparison of mean age with 95% confidence intervals across all included intervention arms demonstrated homogeneity across exercise and surgical intervention arms (see Figure 2).

**Table 3:** Table of description of main aspects of studies

Study	Participants	Intervention arms	Outcome measure
**Exercise**			
**Goren et al. (2010)**	n = 34	1 Exercise and ultrasound	ODI
		2 Exercise and sham ultrasound	
**Koc et al. (2009)**	n = 29	1 Exercise and electrotherapy	RMQ
		2 Exercise and epidural steroid	
		3 Exercise only	
**Malmivaara et al. (2007)**	n = 44	1 Exercise	ODI
**Pua et al. (2007)**	n = 68	1 Treadmill	mODI, RMQ
		2 Cycling	
**Sahin et al. (2009)**	n = 45	1 Exercise	RMQ
		2 Exercise and calcitonin	
**Whitman et al. (2006)**	n = 58	1 Flexion exercise and walking	mODI
		2 Exercise and manual therapy	

**Decompressive surgery**			
**Athiviraham & Yen (2007)**	n = 54	1 Bilateral laminectomy	RMQ
**Cavusoglu et al. (2007)**	n = 50	1 Laminectomy	ODI
**Chopko & Caraway (2010)**	n = 78	1 Minimally invasive decompression	ODI
**Malmivaara et al. (2007)**	n = 50	1 Segmental decompression	ODI
**Sobottke et al. (2010)**	n = 25	1 Minimally invasive decompression	ODI
**Thome et al. (2005)**	n = 40	1 Bilateral laminotomy	RMQ
		2 Unilateral laminotomy	
		3 Laminectomy	
**Weinstein et al. (2008)**	n = 398	1 Laminectomy	mODI
**Yasar et al. (2009)**	n = 125	1 Laminectomy	ODI

ODI = Oswestry Disability Index; mODI = Modified Oswestry Disability Index; RMQ = Roland-Morris Questionnaire

### Types of intervention

#### Exercise for the management of LSS

Flexibility exercises were included in all exercise intervention arms either as part of a formal physiotherapy-supervised or home exercise program. Strengthening exercises were included in nine of the exercise intervention arms [10,25-28]. Specific details of these exercises were not included in any of the studies. Aerobic exercise was included in some studies [24-26] and not others.

Outpatient exercise interventions ranged in duration from 3-weeks [26,28] to 6-weeks [24,25]. One study's exercise intervention [27] was undertaken with inpatients, but all three intervention arms in this study included an ongoing home exercise program. Ten of the 12 exercise intervention arms included a home exercise program. Adherence to and duration of the home exercise programs were poorly described. A wide range of co-interventions was administered in conjunction with exercise, including medication (analgesics, non-steroidal anti-inflammatories), epidural steroid, manual therapy and electrotherapy.

#### Decompressive surgery for the management of LSS

Two studies described a minimally invasive technique for lumbar decompression [30,31], whereas all other surgical studies reported laminectomy via varying approaches. Two intervention arms reported bilateral foraminotomies as part of their surgical approach [23,34] and five intervention arms reported partial medial facetectomies [10,32,34]. Although studies that included subjects with degenerative spondylolisthesis were excluded, three studies reported a proportion of subjects managed with fusion in addition to their decompressive surgery; although reasons for this were not described [10,33,34].

### Effect of exercise compared to decompressive surgery

The study by Malmivaara and colleagues [10] was the only study that directly compared the effect of decompressive surgery and a non-operative intervention that included an exercise component. The study reported a global difference in ODI over 2-years in favour of decompressive surgery (*p *= 0.01). The effect sizes at respective follow-up periods were 0.55 at 6-months, 0.81 at 12-months and 0.56 at 24-months.

**Figure 3:**
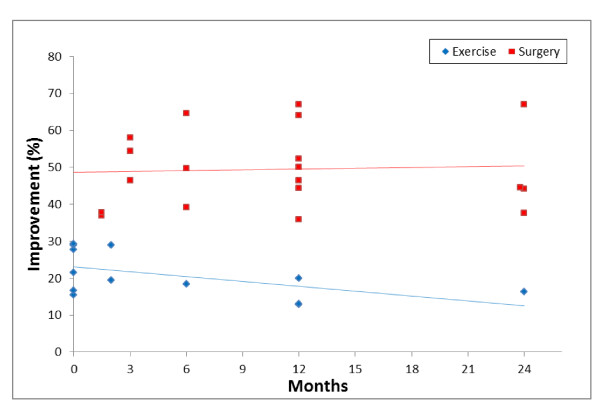
**Percentage change in patient report functional outcome scores (with lines of best fit)**.

Across the 22 intervention arms, baseline and post-intervention data were available for all studies except the three intervention arms in the study by Koc and colleagues [27]. The percentage change in patient reported functional outcome scores was calculated for 32 instances and is shown graphically in Figure 3.

### Effect of exercise

Data was available from five out of the six studies that examined exercise, enabling calculation of percentage change in patient reported functional outcome scores [10,24-26,28]. Eight separate instances from four studies reported an improvement (range 16-29%) in the first 3-months [24-26,28]. At 6-months, only one instance was reported (18%) [10], three at 12-months (13-20%) [10,25] and one at 24-months (16%) [10].

Goren and colleagues [26] was the only study that examined exercise against a control. One intervention arm received exercise and ultrasound, a second received exercise and sham ultrasound, while a third acted as a control. The two exercise intervention arms demonstrated statistically significant differences compared to the control (*p *< 0.05) at the completion of the intervention. Effect sizes for the exercise and ultrasound arm and the exercise and sham ultrasound arm were calculated as 0.04 and 0.44, respectively.

### Effect of decompressive surgery

Data was available for all ten surgical arms, enabling calculation of percentage change in patient reported functional outcome scores. Nineteen instances of outcome reporting were available from 6-weeks to 24-months post intervention.

Five instances reported improvements in patient reported functional outcome scores (range 37-58%) in the first 3-months [29,30,33,34]. At 6-months, there were three instances of reported improvement (39-65%) [10,31,33], seven at 12-months (36-67%) [10,31-34], and four at 24-months (38-67%) [10,23,33,34].

Decompressive surgery was compared to a control in two studies [23,33]. The calculation of effect sizes was only possible in the Weinstein study [33], which favoured the surgical intervention arm at all timeframes: effect size of 0.58 at 6-weeks, 0.77 at 3-months, 0.79 at 6-months, 0.69 at 12-months and 0.63 at 24-months. These results are from the as-treat analysis of randomised and observation cohorts. The intention-to-treat analysis did not demonstrate significant differences between decompressive surgery and control, likely due to the large number of subject cross-overs between groups.

### Adverse effects

Surgery with decompressive laminectomy was associated with adverse events, with an overall complication rate in the largest surgical intervention arm (n = 394) of 12% [33]. The most common complication was intra-operative dural tear, ranging from 3% [29] to 14% [10]. Other complications reported included wound infection, ongoing neural dysfunction and re-operation. It was also apparent that some subjects were no better or worse after decompressive surgery [10]. Detailed reporting of adverse effects was not described for exercise interventions, other than in one study, which noted no complications or side effects [26].

## Discussion

This review found that there is strong evidence for improvement in patient reported functional outcomes in those who undergo decompressive surgery for LSS. There is relative consistency between studies across multiple timeframes with sustained improvements through to 2-years post-surgery. All studies that examined surgical decompression reported statistically significant improvements in favour of surgery, with moderate to large effect sizes in two studies [10,33]. Conversely, there was an overall small initial improvement in patient reported functional outcomes in subjects with LSS who participated in an exercise intervention. These improvements subsided over a 2-year period; however there was limited data for exercise interventions at 2-years.

Given the above findings, it is reasonable to question the use of exercise in the management of patients with LSS. Current guidelines support a trial of conservative management prior to surgery [13]. Malmivaara's study [10] reported that only four of 44 subjects conservatively managed proceeded to surgery, while remaining subjects appeared to self-manage their condition. Weinstein and colleagues [33] also found that the majority of subjects managed without surgery showed small improvements in all outcomes. The selection of exercise for LSS may also be given preference over surgery due to underlying surgical risks, including mortality, particularly in the older population who often present with multiple co-morbidities [35]. Despite the significant and sustained improvements in patient reported functional outcomes shown with decompressive surgery in this systematic review, self-management may still be a worthwhile option prior to consideration of surgical intervention.

A number of issues within included studies may have influenced the results of this systematic review. There were four surgical studies in this review that only included subjects who had failed conservative management [23,29,30,32]. These studies may represent samples with more disabling cases of LSS. This creates the possibility of the surgical outcomes being biased towards subjects who had failed conservative management.

The results from the included exercise studies were limited by the implementation of poor quality interventions. Exercise interventions were likely of inadequate duration to demonstrate change in outcome scores. There was also an absence of tailored exercise towards subjects' individual impairments in all but two studies [10,25]. Subjects' adherence to exercise also varied between studies.

The moderate methodological quality of included studies was also likely to affect the results of this review. Common issues were small sample sizes, lack of sample size calculations, inadequate description of interventions and numerous co-interventions. There appeared to be a discrepancy in mean age between exercise and surgical intervention arms (59.4 and 66.4 years, respectively); however, testing of homogeneity did not demonstrate a significant bias in sampling.

The availability of only two randomised controlled trials limited calculation of effect sizes. More so, there was only one study that directly compared decompressive surgery and exercise for LSS [10]. Ideally, this review would have limited its study inclusion criteria to randomised controlled trials and therefore represent a collation of the highest level of evidence. Given the sparseness of high level evidence in this topic, the methodology of this systematic review was modified to include lower levels of evidence. This systematic review of current best available evidence is therefore able to provide useful information to inform clinical practice and future research.

### Implications for clinical practice

Due to the heterogeneity of the land based exercise interventions and numerous co-interventions reported in the included literature, this systematic review is not able to provide guidance as to whether certain types of exercise (mode, intensity, duration, location) are more effective in managing patients with LSS. It is however apparent from the randomised clinical trials that investigated numerous exercise interventions that there were statistically significant improvements (*p *< 0.05) within each group relative to baseline, but no significant differences between groups when different interventions were compared. The authors would suggest that the literature therefore supports a broad approach to exercise interventions rather than supporting a particular exercise type. This review supports the findings of previously reported literature that a trial of conservative management with land based exercise be considered [10,13].

### Implications for research

Further research of land based exercise interventions for LSS would benefit from more accurate descriptions of intervention, including type, duration and intensity. Co-interventions should also be minimised. Future research should also continue to embrace the use of reliable and valid outcome measures.

More consistent follow-up through to 2-years would provide valuable insights as to whether the smaller initial gains reported with exercise are maintained. Analysis of subgroups within exercise interventions as to predictors of good and poor response, such as outcome measure score, age or walking distance, would assist with understanding which LSS patients may more readily benefit from land based exercises. Understanding of subgroups may also assist in determining patients that may benefit from other management options, including surgery.

Ideally further research in this patient group would directly compare decompressive surgery and conservative management with land based exercise, with the addition of a control group. However, as demonstrated by Weinstein's review [33], the challenges of implementing a randomised controlled study design of sufficient power, with long term follow-up is limited by both subject non-adherence to randomisation and the ethical considerations around use of a true control group.

## Conclusions

Decompressive surgery is more effective (larger treatment effect) in the management of LSS than land based exercise; however, whilst patients wait for surgery and given the risks of surgery, there are potential benefits in functional improvements from land-based exercise interventions. A self-management program with a land based exercise intervention prior to consideration of surgical intervention for patients with LSS is supported.

Due to the significant heterogeneity of the land-based exercise interventions investigated in the included studies, this systematic review is unable to provide any specific recommendations regarding the most effective forms of exercise.

## Competing interests

The authors declare that they have no competing interests.

## Authors' contributions

MJ and JO worked together to conceptualise the topic, devise the search strategy, carry out the initial search and assess inclusion of studies into review. Quality of studies was independently assessed by MJ and JO. Data extraction for the characteristics of interventions was done by MJ and JO. MJ and JO contributed equally to the initial draft. KGS together with MJ and JO were equally involved in the editing and review of the manuscript. All authors read and approved the final manuscript.

## Pre-publication history

The pre-publication history for this paper can be accessed here:

http://www.biomedcentral.com/1471-2474/13/30/prepub

## Supplementary Material

Additional file 1 **Studies excluded from the review**. Table summarising studies excluded from the systematic review and the reasons for exclusion. Click here for file
