# Supplementary material for: The effectiveness of land based exercise compared to decompressive surgery in the management of lumbar spinal-canal stenosis: a systematic review
Source: BMC Musculoskelet Disord. 2012 Feb 28;13:30. doi: 10.1186/1471-2474-13-30 (PMC3305601; doi:10.1186/1471-2474-13-30)
Supplement: Additional file 1 — Studies excluded from the review. Table summarising studies excluded from the systematic review and the reasons for exclusion. [file 1471-2474-13-30-S1.DOC]

| **Studies** | **Reason for exclusion** |
| --- | --- |
| Amundsen et al. 2000 | No valid patient reported functional outcome measure |
| Cho et al. 2007 | No valid patient reported functional outcome measure |
| Fu et al. 2008 | Population included congenital stenosis |
| Galiano et al. 2005 | Observational study |
| Greenman 2006 | Case series |
| Gunzburg et al. 2003 | Population included congenital stenosis |
| Haro et al. 2008 | No formal diagnosis of stenosis for included subjects |
| Hatta et al. 2009 | Inconsistent follow-up period between subjects |
| Jansson et al. 2009 | No valid patient reported functional outcome measure |
| Jayaroa & Chin 2010 | Retrospective study |
| Kim et al. 2007 | Retrospective study |
| Kleinstuck et al. 2009 | No valid patient reported functional outcome measure |
| Mariconda et al. 2002 | No valid patient reported functional outcome measure |
| McGregor & Hughes 2002a | Population included foraminal stenosis |
| McGregor & Hughes 2002b | Population included foarminal stenosis |
| Murphy et al. 2006 | Population included foraminal stenosis |
| Ng et al. 2007 | Population included spondylolisthesis |
| Orpen et al. 2010 | Recording outcomes >2 years |
| Paker et al. 2005 | No valid patient reported functional outcome measure |
| Pao et al. 2009 | Inconsistent follow-up period between subjects |
| Papavero et al. 2009 | No valid patient reported functional outcome measure |
| Rampersaud et al. 2008 | No valid patient reported functional outcome measure |
| Rosen et al. 2007 | Retrospective study |
| Schillberg & Nystrom 2000 | No valid patient reported functional outcome measure |
| Shabat et al. 2007 | No valid patient reported functional outcome measure |
| Shabat et al. 2008 | Recording outcomes >2 years |
| Sinikallio et al. 2006 | Surgical details not specified |
| Sinikallio et al. 2007 | Surgical details not specified |
| Sinikallio et al. 2009 | Surgical details not specified |
| Sinikallio et al. 2010 | Surgical details not specified |
| Sirvanci et al. 2008 | Retrospective study |
| Spratt et al. 2004 | Observational study |
| Tenhula et al. 2000 | Recording outcomes >2 years |
| Waikakul & Waikakul 2000 | Language other than English |
| Westergaard et al. 2009 | Observational study |
| Whitman et al. 2003 | Case series |
| Wilby et al. 2006 | Population included spondylolisthesis |
| Willen et al. 2008 | Observational study |
| Wu et al. 2003 | Language other than English |
| Yagi et al. 2009 | No valid patient reported functional outcome measure |
| Yaksi et al. 2007 | No valid patient reported functional outcome measure |
| Yamashita et al. 2006 | Recording outcomes >2 years |
| Yukawa et al. 2002 | Population included spondylolisthesis |

**References**
